# A Classifier Graph Based Recurring Concept Detection and Prediction Approach

**DOI:** 10.1155/2018/4276291

**Published:** 2018-06-07

**Authors:** Yange Sun, Zhihai Wang, Yang Bai, Honghua Dai, Saeid Nahavandi

**Affiliations:** ^1^School of Computer and Information Technology, Beijing Jiaotong University, Beijing 100044, China; ^2^School of Computer and Information Technology, Xinyang Normal University, Xinyang 464000, China; ^3^Deakin University, Melbourne, VIC 3125, Australia; ^4^Institute for Intelligent Systems Research and Innovation, Deakin University, Waurn Ponds, VIC 3220, Australia

## Abstract

It is common in real-world data streams that previously seen concepts will reappear, which suggests a unique kind of concept drift, known as recurring concepts. Unfortunately, most of existing algorithms do not take full account of this case. Motivated by this challenge, a novel paradigm was proposed for capturing and exploiting recurring concepts in data streams. It not only incorporates a distribution-based change detector for handling concept drift but also captures recurring concept by storing recurring concepts in a classifier graph. The possibility of detecting recurring drifts allows reusing previously learnt models and enhancing the overall learning performance. Extensive experiments on both synthetic and real-world data streams reveal that the approach performs significantly better than the state-of-the-art algorithms, especially when concepts reappear.

## 1. Introduction

In recent years, with the technological advance, a growing number of applications produce large amounts of data streams at high speed. Examples include sensor networks, spam filtering systems, traffic control, and intrusion detection [1, 2]. More formally, a data stream* S* is a potentially unbounded, ordered sequence of instances, which arrive continuously at high-speeds.

One of the biggest challenges in data stream learning is to deal with concept drift [3–5], i.e., the underlying concept may drift dynamically over time. Concept drift frequently occurs in the real world. For example, in recommend systems, user consumption preferences may change over time due to fashion, economy, and so on and weather prediction models may change according to the seasons. Such changes lead to a drastic drop in classification performance. A reasonably useful classifier should have the capability to recognize and respond to such changes accordingly and accurately. This study focuses on the topic of developing classifier learning systems for mining data streams in dynamic environments.

Concept drift can be divided depending on their speed, into sudden and gradual drifts [4]. Sudden concept drift is characterized by large amounts of changes between the underlying class distribution and the incoming instances in a relatively short amount of time. Gradual concept drift can take a very large amount of time to see a significant change in differences of underlying class distributions between the old instances and the incoming instances. In fact, no matter what type of change occurs, the model should be able to track and adapt to changes accordingly.

It is common that in real-world data streams previously seen concepts may reappear in the future. For example, weather prediction models change according to the seasons and a popular topic may appear in a social network during the time of the year (i.e., festivals or elections) [4, 6]. This demonstrates a unique kind of drift, known as recurring concepts [6]. For example, news reading preference of a user may change over time. A user can have different choices on mornings, evenings, weekdays, and weekends. In addition, a user might search for astrology articles in the beginning of the year and financial articles at the beginning of each quarter. Unfortunately, only few approaches take recurring concepts into consideration [6–8]. In the situation where concept may recur, the classification models which have been used in the past may apply to the future use. However, most existing papers on drift detection neglect this phenomenon and tend to take the concept which occurs after a drift as a new one.

If a concept reappears, the previously learnt classifiers should be reapplied; thus the performance of the learning algorithm can be improved. This study focuses on the problem of recurring concepts. Two crucial problems will be addressed: (1) How to detect the recurring concepts. (2) How to effectively adapt when a recurring concept is detected.

Due to the fact that drift detection can capture changes in data streams timely and then update the predictive model, the predictive model can maintain high accuracy. Following these critical motivations, an efficient scheme is designed to deal with the above issues. The key contribution of our algorithm is threefold:

(1) A Drift Detection Method named Distribution-Based Detection Method (DBDM) is introduced aiming at addressing the first issue. It detects changes by comparing the distribution of data in different time windows based on Bernstein inequality.

(2) An algorithm named the Recurrent Detection and Prediction (RDP) approach is introduced targeting on solving the second problem. It stores concepts which present previously occurred concepts with a graph model.

(3) The performance comparison results of the proposed algorithms evaluated on a variety of datasets demonstrated that our method is both stable enough on the data streams with gradual concept drifts and flexible enough to adapt effectively to sudden concept drifts and the recurring concepts problem.

The rest of the paper is as follows.  Section 2 reviews the related work. In Section 3, we discussed the basic ideas of the presented algorithms in detail.  Section 4 provided the experimental results tested on both real and synthetic datasets followed by analysis and discussions. In the last section, we draw conclusions and discussed future work.

## 2. Related Work

In this section, first, some relevant concepts will be introduced, then, several related algorithms will be reviewed, and finally, based on these previous works, the original contributions of the paper will be summarized.

### 2.1. Basic Concepts and Notations

**Definition 1 (data streams).**

Let **S** be an infinite* d*-dimensional data stream

(1) $$\begin{array}{c} S=\left\{\;\left(\;x_{1},y_{1}\;\right),\left(\;x_{2},y_{2}\;\right),\ldots ,\left(\;x_{t},y_{t}\;\right),\ldots \;\right\}. \end{array}$$

Each instance is a pair (*x*_*t*_,* y*_*t*_), where* x*_*t*_ is a vector of attribute values arriving at the time stamp* t* and* y*_*t*_ is the class label of* x*_*t*_.

**Definition 2 (concept drift).**

The term concept drift can be formally defined as any scenario where the joint probability, which represents data distribution, changes over time, i.e., *P*_*t*_(*x*_*i*_, *y*_*i*_) ≠ *P*_*t*+1_(*x*_*i*_, *y*_*i*_) [9].

**Definition 3 (sudden concept drift).**

**Figure 1:**
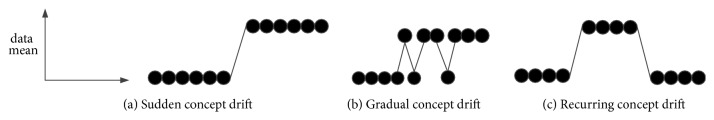


As shown in Figure 1(a), sudden concept drift means that the distribution of data will be changed directly to a new one in a relatively short time.

**Definition 4 (gradual concept drift).**

As shown in Figure 1(b), gradual concept drift means that the probability of the old data distribution will decrease and the probability of a new distribution will increase during a very large amount of time to see a significant change.

**Definition 5 (recurring concept drift).**

As shown in Figure 1(c), a recurring concept drift occurs when the instances from a period* k* are generated from the same distribution as a previously observed period* P*_*k*_(*x*_*i*_,* y*_*i*_) = *P*_*k*-*j*_(*x*_*i*_,* y*_*i*_).

**Definition 6 (classifier graph).**

A classifier graph is a graph whose nodes are stored with distinct classifiers derived from a given stream data. Each node in a classifier graph stores a classifier; directed link represents a drift; weight of a link represents drifting times.

### 2.2. Handling Concept Drift in Data Streams

Approaches to cope with concept drift can be divided into two main categories: passive and active approaches [5, 10]. The first approach adapts a learner at regular intervals without considering whether changes have really occurred, and it tracks changes blindly and updates the model continuously without requiring explicit change detection, while the second approach only makes adjustment when a drift occurs.

Active approaches typically require change detection modules. Gama et al. [11] presented a Drift Detection Method (DDM) which detects change by monitoring the classification error rate. Baena-Garcia et al. [12] introduced a detection algorithm called Early Drift Detection Method (EDDM) which has a better performance in the scenario of gradual change. Adaptive Windowing (ADWIN) [13] adopted a sliding window to store instances recently read and divides the window into two subwindows to monitor changes in the subwindows. EWMA for Concept Drift Detection (ECDD) was introduced by Ross et al. [14], which uses exponentially weighted moving average charts to monitor the error rate. Sakthithasan et al. [15] proposed an algorithm named SeqDrift which adopts reservoir sampling to manage the memory and improve the detection sensitivity in the case of slow gradual change.

Ensemble classifiers are one of the most popular passive approaches, and many active approaches based on ensemble are available in the literature. Streaming Ensemble Algorithm (SEA) [16] is one of the earliest solutions which adopt an ensemble strategy to address concept drift. It makes final prediction using a simple majority voting. Accuracy Weighted Ensemble (AWE) [17] is similar to SEA. It decides the weight of base leaner according to the classification accuracy of the learner on test datasets. Dynamic Weighted Majority algorithm (DWM) [18] is an ensemble based on the weighted majority mechanism and each classifier corresponds to a weight which can change dynamically. Online Coordinate Boosting (OCBoost) [19] is an online boosting algorithm, which adopts online boosting strategy to achieve a better performance. Diversity for Dealing with Drifts (DDD) [20] is a novel ensemble method which maintains ensembles with different levels of diversity.

While active approaches, such as change detection, work quite well in coping with sudden concept drift, passive approaches work better for gradual drift which is nevertheless more difficult to detect. In order to react to different types of concept drifts immediately, some approaches try to utilize both passive and active techniques to aid learning in nonstationary environments, which seek to combine the best elements of both passive approaches and active approaches.

As we pointed out earlier it is very common in real-world data streams that previously seen concepts will reappear. This suggests a unique kind of concept drift, known as recurring concepts. However, none of the aforementioned approaches take the problem of Recurring Concept Drifts into account. The concept of Recurring Concept Drifts has been introduced by Widmer et al. In [8], Katakis et al. presented a framework to detect recurring contexts. In this framework, batches of instances are mapped into conceptual vectors and stream clustering is used to group these conceptual vectors into different clusters. Then incremental classifiers are trained for every cluster. When a new batch of instances arrives, it was assigned into an existing cluster or a new cluster is created for it. Gomes et al. [21] introduced a data stream learning system which exploited context information to add and delete classifiers in the ensemble to improve existing ensemble approaches to deal with concept drift and recurring concept. Abad et al. [22] devised a framework to solve the issue of recurring concept in spam filtering. The framework handles the context which is related to concept drift. Gonçalves et al. [23] presented a framework named Recurring Concept Drifts (RCD) to handle recurring concept drift. In the framework, the new classifier is created when new concept occurs and a group of instances corresponding to the new concept is stored. When a new drift is detected, RCD compares the incoming instances with the previous ones to validate whether the two sets of instances come from the same distribution. If they are from the same distribution, the previous classifier is reused.

## 3. Learning Recurring Concept from Classifier Graph

In this section, a novel change detection paradigm based on the distribution between two subwindows will be introduced first, and then an internal classifier graph empowered drift detection mechanism will be presented in detail.

### 3.1. Change Detection Problem

Let* W*_1_= (*x*_*t*+1_,…,* x*_*t*+*n*_) denote reference window, let* W*_2_ = (*x*_*t*+*n*+1_,…,* x*_*t*+2*n*_) represent current window, let ${\hat{\mu }}_{W_{1}}$ and ${\hat{\mu }}_{W_{2}}$ be the average value of* W*_1_ and* W*_2_, and let* μ*_*W*1_ and* μ*_*W*2_ be their expected value. In fact, the underlying distribution of data is unknown and a test statistic based on sample means needs to be adopted by the change detector. This is accomplished by carrying out statistical tests that verifies whether the classification error or class distribution remains constant over time. The problem of change detection in data streams is to determine the null hypothesis* H*_0_ against alternative hypothesis* H*_1_ as follows:

(2) $$\begin{array}{c} H_{0}\;\mathrm{Pr}\left(\left|{\hat{\mu }}_{W_{1}},{\hat{\mu }}_{W_{2}}\right|\geq \varepsilon \right)\leq \delta \\ H_{1}\;\mathrm{Pr}\left(\left|{\hat{\mu }}_{W_{1}},{\hat{\mu }}_{W_{2}}\right|\geq \varepsilon \right)>\delta \end{array}$$

where *δ* ∈ (0,1) is a confidence value, while *ε* is a function of* δ* when test statistics are used to model the difference between the means of instance in two windows. The change detection raises a change alarm, when the difference is greater than a threshold.

### 3.2. Distribution-Based Change Detection Using Bernstein Inequality

In this paper, a Distribution-Based Detection Method (DBDM) was presented by comparing the distribution of data in different time windows. The two-window paradigm is exploited by comparing error rate of the classifiers extracted from old and recent data. A correct prediction can be considered to be 1 and incorrect prediction to be 0. In the change detection, windows contain simple information (bits or numbers); thus the change tests are really simple. The sliding window* W* was partitioned into two equal length subwindows: a left subwindow* W*_1_ and a right subwindow* W*_2_ with means* μ*_1_ and* μ*_2_, respectively. Whenever the amount of new data reaches* m*, the boundary between the new data and the data arrived before it is taken as a check point. Then every check point is traversed to check whether the difference between the mean value of the data in the left subwindow of the check point and that of the right subwindow is greater than a threshold.

The key to the detection method is the calculation of the threshold*ε*. The inequalities which are often used to depict the difference between two distributions are Hoeffding inequality [24], Chernoff inequality [25], and Bernstein inequality [26]. Among them, the Hoeffding inequality is widely used in previous research. However, the Hoeffding inequality neglects the effect of variance, which leads to imprecise results in the case of small variance. Bernstein inequality associates expected value with variance. For this reason, a more precise threshold can be obtained by using Bernstein inequality. It is defined as follows:

(3) Pr⁡1n∑i=1nXi−EX≥ε≤2exp⁡−nε22σ2+2/3εc−a

where *X*_1_, *X*_2_, ···, *X*_*n*_ are independent random variables, *X*_*i*_ ∈ [*a*, *c*], *EX* is the expected value, and *σ*^2^ is the variance.

The probability of capturing concept drift at a certain check point is no more than *δ*, which can be expressed as shown in

(4) $$\begin{array}{c} \mathrm{Pr}\left[\;\left|\;{\hat{\mu }}_{1}-{\hat{\mu }}_{2}\;\right|\geq \varepsilon \;\right]\leq \delta \end{array}$$

Applying Boole's inequality, (4) can be converted into

(5) Pr⁡μ^1−μ^2≥ε≤Pr⁡μ^1−μ≥kε+Pr⁡μ^2−μ≥1−kε

where* k* is the proportion of data among the left and right side.

Apply Bernstein inequality on the RHS of (5):

(6) Pr⁡μ^1−μ^2≥ε≤2exp⁡−n1kε22σ12+2/3kεc−a+2exp⁡−n21−kε22σ22+2/31−kεc−a

where *σ*_1_^2^ and *σ*_2_^2^ represent variances. By substituting* a* and* c* with 0 and 1 in (6), we obtain

(7) Pr⁡μ^1−μ^2≥ε≤2exp⁡−n1kε22σ12+2/3kε+2exp⁡−n21−kε22σ22+2/31−kε=δ′

It is worth noting that formulation of the optimization problem for determining* k* is based on asymptotic behavior and so that* k*'s value is approximate. Equating the two terms in expression yields

(8) $$\begin{array}{c} n_{1}\sigma_{1}=n_{2}\sigma_{2} \end{array}$$

Let *δ*′ in (7) be equated to the user-assigned *δ* and set the two exponents to be equal. We have

(9) δ=4exp⁡−n11−kε22σ12+2/31−kε=4exp⁡−n11−kε22n1σ12/n2+2/31−kε

We have equated *δ* to the left exponential term in (7) instead of the right one to get *ε*. We have

(10) $$\begin{array}{c} \varepsilon =\frac{1}{3n_{1}k}\left(\;\ln\frac{4}{\delta }+\sqrt{{\left(\ln\frac{4}{\delta }\right)}^{2}+18n_{1}{\sigma_{1}}^{2}\ln\frac{4}{\delta }}\;\right) \end{array}$$

We set *δ*_*warning*_ and *δ*_*drift*_, and then *ε*_*warning*_ and *ε*_*drift*_ can be calculated. When the difference of the mean values is greater than *ε*_*warning*_, meaning that a drift has been triggered. And when difference of the mean values is greater than *ε*_*drift*_, we confirm that the concept has been changed.

In DBDM, before an actual change point is detected multiple check points need to be detected. In multiple testing problems, as the number of hypotheses being tested increases, the likelihood of incorrectly rejecting a null hypothesis increases. Most of the existing algorithms adopt Bonferroni correction [27] to avoid the problem. However, Bonferroni correction is less effective when there are a large number of tests. For this reason, we adopt the error correction factor based on Šidák correction [28]. *δ*_*s*_ is modified according to

(11) $$\begin{array}{c} \delta_{s}=1-{\left(\;1-\delta \;\right)}^{1/\sqrt{n}} \end{array}$$

where* n* denotes the number of hypotheses. The pseudocode is presented in Algorithm 1.

### 3.3. Recurrent Detection and Prediction Approach

In this section, we design a novel triggered rebuild approach named Recurrent Detection and Prediction (RDP) approach that uses a weighted directed graph to predict which previously occurred concept is the most likely to recur when a new concept drift is detected.

In RDP, the recurring concepts and corresponding instances will be stored. Directions and weights of the arrows indicate the transformation relation of the concepts stored in graph. Whenever the change detection reaches the warning level, a set of instances will be stored and a new classifier will be trained. When the state is transformed into drift, verification will be carried out to see whether the latest stored instances and instances which have been stored in graph are same. If they are drawn from the same distribution, the current concept will be regarded as a recurring concept. Then the stored instances in classifier will be utilized in order to replace the current classifier. Otherwise, we regard that a concept has occurred. Then a new classifier is trained and the set of instances are in a graph.

**Figure 2:**
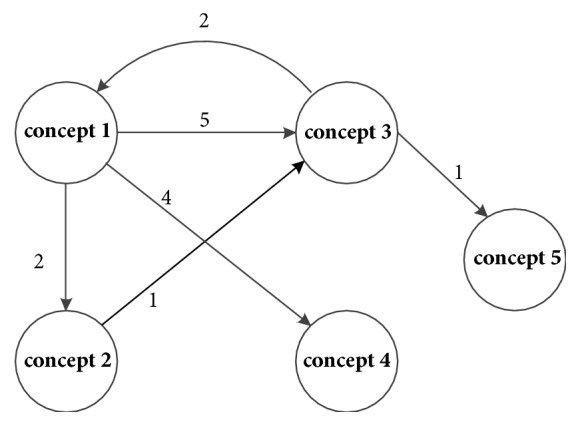
Classifier graph diagram.

**Figure 3:**
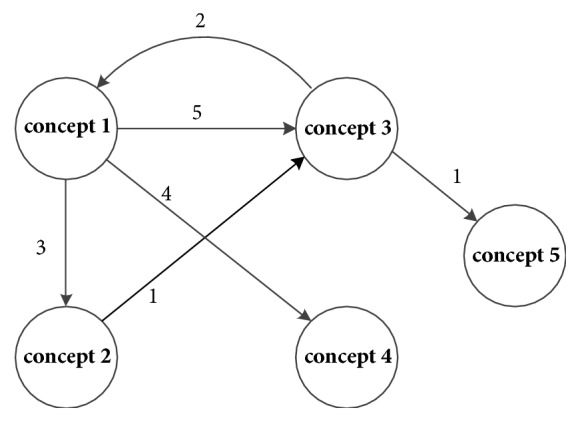
Concept 2 recurs.

**Figure 4:**
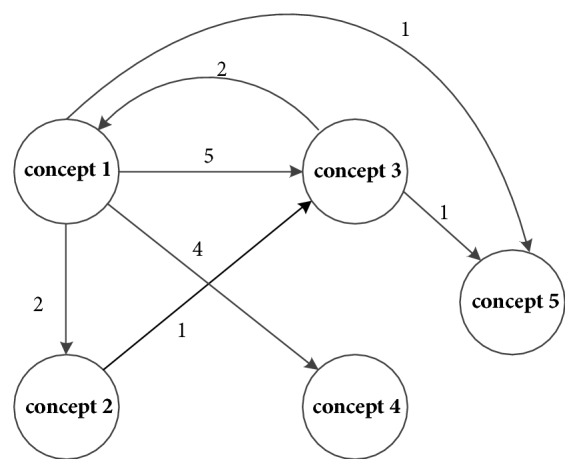
Concept 5 recurs.

**Figure 5:**
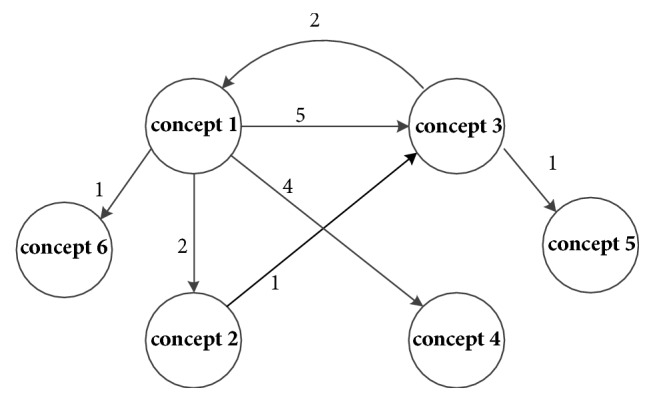
New concept occurs.

As defined in classifier graph, each node stores a classifier and a set of instances used to induce the classifier. When concept drifts occur, one will be added to the weight of the arrow whose to-node is the new concept and the from-node is the concept occurring before the drift. And next time when a drift is detected, classifier graph can be employed to predict which previously occurred concept is the most likely to recur this time. The algorithm is illustrated in detail with an example as Figures 2–5.

As shown in Figure 2, provided that the current concept is concept 1, we can detect concept drift after a period of time. Because the arrow directed from concept 1 to concept 3 has the maximum weight among the arrows whose from-node is concept 1, we assume that concept 3 recurs this time. If it is false, we choose the arrow whose weight is the second maximum. The procedure will be repeated. If all arrows whose from-node is concept 1 which does not recur this time, then the node which is not the one of to-node of concept 1, i.e., concept 5, will be checked to see whether it is a recurring concept. There are three cases:

(1) Concept 2, concept 3, or concept 4 recurs this time. We assume that concept 2 recurs, and then we add the weight of the arrow from concept 1 to concept 2, as shown in Figure 3.

(2) As shown in Figure 4, concept 5 is the recurring concept. An arrow will be added from concept 1 to concept 5.

(3) When a new concept 6 occurs, a node will be created and an arrow will be added from concept 1 to concept 6, as shown in Figure 5.

Let* G* represent the graph,* V* is the set of vertexes in* G*,* vexnum* denotes the serial number of vertexes in* G*,* C*_*n*_ is a new classifier which applies to the new concept,* V*_*k*_.*C* represents the previous classifier of* V*_*k*_.* B*_*n*_ represents a set of instances which corresponding to new concept, and* p* is the order of the old concept in* G*. The pseudocode of RDP is listed in Algorithm 2.

## 4. Experiment Results and Analysis

The algorithms were carried out with help of MOA (Massive Online Analysis) (Version: MOA Release 2017.7) [29]. MOA is a software environment for implementing algorithms and running experiments for online learning. All of the experiments were performed on an Intel Core i3-2120 CPU @ 3.3GHz with 8GB of RAM running Windows 7.

To evaluate the performance of the proposed algorithms, a series of experiments were carried out. Two main sets of experiments are presented:

Experiment 1 aims to verify the fact that the performance of the proposed change detection in different drift scenarios.

Experiment 2 is set to compare the performance of the RDP with other state-of-the-art approaches.

### 4.1. The Analysis of DBDM

In the first experiment, the performance of DBDM is compared against the following algorithms: DDM [11], EDDM [12], and ECDD [14] in terms of the false positive counts. In DBDM, we set *δ*_*drift*_ = 0.1, *δ*_*drift*_ = 0.1 and the size of block is 500. In ECDD, we set* ARL*_0_=1,000 and *λ* = 0.2.

We generated four datasets. Each has 200,000 instances which were drawn from a stationary Bernoulli distribution and the mean value of the Bernoulli distribution is set to 0.05, 0.1, 0.3, and 0.5, respectively [13]. To make the experimental more reliable, the algorithms were carried out on each dataset for 10 times and then calculated the mean.

Table 1 shows that the false positive count of DBDM is lower than EDDM and ECDD, but it is higher than DDM. It also demonstrates that the false positive count of DBDM is reduced from 3.39 to 0.03 with the increase of the mean value of Bernoulli distribution.

Then, we compared the performance of detectors under sudden concept drift scenario. We generated four datasets of 200,000 instances drawn from a Bernoulli distribution. The first 100,000 instances were drawn from stationary Bernoulli distribution whose mean value was 0.01. And the mean of the last 100,000 instances was raised to 0.05, 0.1, 0.3, and 0.5 for the four datasets separately.

Table 2 shows that the false detection of DBDM is the lowest when the change of the mean is small. However, the false positive rate of DBDM is increased with the increase of the mean value.

Table 3 shows the average detection delays under sudden concept drift. In general, the detection delays decrease with the increase of the mean value. The detection delay of DDM is higher when the mean value increment is low, and then it decreases rapidly. The detection delay of EDDM is high when the mean value increment is 0.04, and then it decreases gradually. At the beginning, the delay of ECDD is lower than DDM and EDDM, and then it drops slightly. However, the delay of ECDD increases when the mean value of increment is 0.29. The detection delay of DBDM is lower and it has a downward trend. This is due to the fact that the management of the recurrent change detection mechanism is capable of reusing previous concepts and gains the better performance in different situations, particularly under concept drift environments.

Finally, we investigated the performances of the four detection methods in the scenario of gradual change. When drift happens incrementally, the change is not obvious. Therefore, we mainly focus on detection delay and false negative. Detection delay is the distance between the instance at which the change is detected and the instance at which the change really occurs. In the experiment, we generate a dataset contains 1,000,000 instances. The first 998,000 instances are stable, and the mean value is 0.01. Then mean values of the last 2,000 instances of the four datasets rise with a different slope separately. The slopes are 0.0001, 0.0002, 0.0003, and 0.0004. The false negative counts are as shown in Table 4. The false negative counts of ECDD and DBDM are 0. And the false negative counts of DDM and EDDM decrease with the increase of the slope.

The detection delays are demonstrated in Table 5. It can be seen that DDM and EDDM are not good at detecting gradual drift. ECDD is rather high, although it reduces a little with the increase of the slope. DBDM is high at the beginning, but it reduces obviously with the increase of the slope. It demonstrates that DBDM is superior to other methods. It is partly because the management of the recurrent change detection mechanism is capable of reusing historical concepts and achieving the better performance in this scenario.

### 4.2. Comparative Performance Study

This part demonstrates the experimental results with regard to the effectiveness and efficiency of the proposed method.

#### 4.2.1. Datasets

In the second experiment, we adopted four synthetic and five real-world datasets. The datasets are summarized in Table 6.

***Synthetic Datasets*. **In the experiment, the synthetic datasets contain three types of concept drift: gradual, sudden, and recurring concept drift.

HyperPlane dataset is represented by the set of points* x* that satisfy ∑_*i*=1_^*d*^*w*_*i*_*x*_*i*_ = *w*_0_, where* x*_i_ is the* i*th coordinate of* x*. Two classes are distinguishing in the following way: instances for which ∑_*i*=1_^*d*^*w*_*i*_*x*_*i*_ ≥ *w*_0_ are labeled positive and instances for which ∑_*i*=1_^*d*^*w*_*i*_*x*_*i*_ < *w*_0_ are labeled negative. Drifts was introduced by changing each weight attribute *w*_*i*_ = *w*_*i*_ +* dσ*, where* σ *is the probability that the direction of change is reversed and* d* is the change applied to every instance. This generator was adopted to create a dataset contains 1,000,000 instances with gradual drifts by the modification weight *w*_*i*_ changing by 0.001 with each instance, and 5% noise was added to streams.

LED dataset is to predict the digit displayed on a seven-segment LED display. The particular configuration of the generator used for the experiment produces 24 binary attributes, 17 of which are irrelevant. Concept drift is simulated by interchanging relevant attributes. We generated a stream of 1,000,000 instances with sudden concept drifts and 15% of noise.

Random Tree dataset is generated by Random Tree generator and it contains 1,000,000 instances and 10 attributes. It has four recurring concepts which evenly distributed throughout the 1,000,000 instances.

SEA dataset consists of three attributes, where only two are a relevant attributes. All three attributes have values between 0 and 10. The points of the dataset are divided into four blocks with different concepts. In each block, the classification is done using* f*_1_ +* f*_2_ ≤ *θ*, where f1 and f2 represent the first two attributes and *θ* is a threshold value. The most frequent values are 9, 8, 7, and 9.5 for the data blocks. It contains 1,000,000 instances with sudden drifts reappearing every 250,000 instances and 10% of noise.

***Real-World Datasets*.** Real-world stream environment conceptual changes have unpredictability and uncertainty which can better verify the performance of the algorithm.

Emailing list (Elist) contains a stream of emails on various topics which are shown to the user one after another and are marked as interesting or junk. It is composed of 1, 500 instances with 913 attributes and is divided into 5 periods every 300 instances. At the end of each period, the user's interest in a topic changes in order to simulate the occurrence of concept drift. Thus, the transformation between two periods is a signal of drift. The dataset can be obtained at http://mlkd.csd.auth.gr/concept_drift.html. The characteristics of Elist are presented in Table 7, where (+) means an interested email and (-* *-) indicates a spam.

Spam dataset represents the scenario of gradual concept drift and is based on the Spam Assassin Collection [8] available in http://spamassassin.apache.org/. It consists of 9, 324 instances with 500 attributes.

Usenet dataset simulates a news filtering system with the presence of concept drifts relative to the change of interest of a user over time [8]. The dataset contains 5,931 instances representing documents collected from the 20 Newsgroups. It is available at http://www.liaad.up.pt/kdus/products/datasets-for-concept-drift.

Covertype dataset from UCI archive [30] contains 581,012 instances, 54 attributes, and no missing values. The aim is to predict the forest cover type based on cartographic variables. It can be obtained at http://moa.cms.waikato.ac.nz/datasets/, and then we simulated the dataset into streams by the MOA generators.

Gas Sensor Drift Dataset also from UCI archive contains 13,910 measurements from 16 chemical sensors utilized in simulations for drift compensation in a discrimination task of 6 gases at various levels of concentrations. The dataset was gathered within January 2007 to February 2011 (36 months) in a gas delivery platform facility situated at the Chemo Signals Laboratory in the Bio Circuits Institute, University of California San Diego.

#### 4.2.2. Comparative Study

We compared RDP with five ensemble-based methods: Accuracy Weighted Ensemble (AWE), Ensemble Building (EB), Dynamic Weighted Majority (DWM), Online Coordinate Boosting (OCBoost), and Recurring Concept Drifts (RCD). AWE is the best-known representative of block-based ensembles for data streams. Similar to AWE, EB constructs a subset of classifiers from sequential data chunks and then are used in the ensemble. DWM is based on the weighted majority algorithm, which maintains an ensemble of classifiers and each classifier corresponds to a weight which can change dynamically. OCBoost is an online boosting algorithm. RCD is a framework to deal with recurring concept drift.

The performance of the analyzed algorithms can be evaluated with respect to accuracy, time efficiency, memory usage, and F_1_-measure. The results are shown in Tables 8–11. F_1_-measure represents a harmonic mean between recall and precision. The calculation equation is as follows:

(12) $$\begin{array}{c} F_{1}=\frac{2\times Re\;call\times Pr\;ecision}{Re\;call+Pr\;ecision} \end{array}$$

(1) In terms of accuracy, as shown in Table 8, RDP makes the overall best performance on most of datasets. Specifically, RDP demonstrates significantly the best results on the data steams with recurrent concept drift (Random Tree and Elist). On the dataset with gradual concept drift (HyperPlane), the block-based ensemble AWE is the best, followed by EB. Moreover, DWM seems to be the most accurate in the streams with sudden changes (LED and SEA). On the real-world datasets (Covertype and Gas Sensor), RDP clearly outperformed the other algorithms. For all of datasets, RDP is able to significantly boost the performance by using the recurrent change detection.

(2) Concerning run time, as expected, online classifier like OCBoost requires the most time for classification, following by DWM, and RDP is the least time-consuming. This is partly because the addition of change detection mechanism offers quicker reactions to sudden and recurring concept drift compared to other methods. For this reason, RDP is able to capture changes much more efficiently and adapt to different kind of drifts immediately. Also, notice that AWE and EB did not perform as well as RDP due to the fact that they had to wait to accumulate instances into a batch before learning.

(3) Analyzing the values in Table 10, it can be observed that online ensemble DWM and OCBoost require the least memory storage, following by RDP. It is due to the fact that DWM and OCBoost only update weights of classifiers after each incoming instance without storing data. The memory consumption of AWE and EB is more than RCD and RDP. It is partly because of the fact that RCD and RDP maintain a pool of historical concepts which are checked for reuse. For RDP, we may observe a marginal increase in memory consumption, due to the need of storing and processing weights assigned to classifiers. However, the additional cost is practically negligible.

(4) In terms of F_1_-measure, as shown in Table 11, RDP obtains the overall best performance on most of datasets, followed by RCD, and OCBoost is the worst. This is partly because the management of the recurrent change detection mechanism is capable of reusing previous concepts and gains the better performance in different situations, particularly under concept drift environments. However, other ensemble methods lack detection mechanisms and therefore adapt ineffectively to drifts.

**Figure 6:**
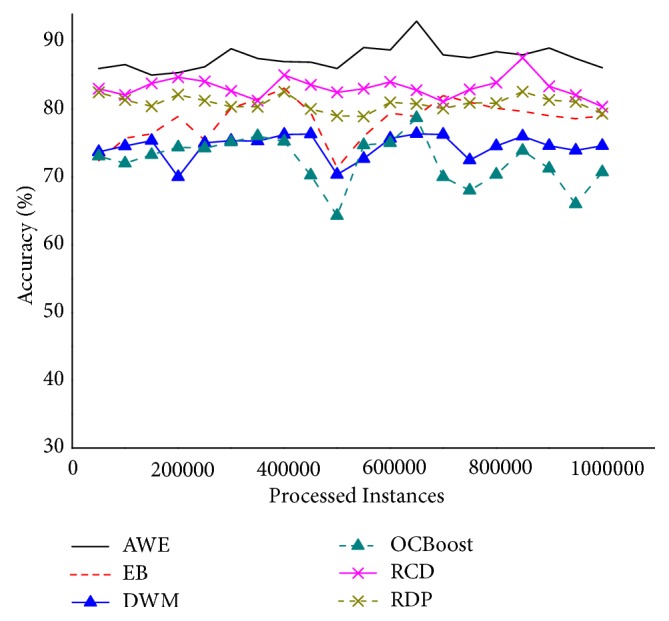
Accuracy on the HyperPlane.

Figure 6 shows the accuracy on the HyperPlane, which is devised to evaluate the ability to handle gradual drifts. It is found from Figure 6 that the trend of all algorithms is basically the same. Among them, AWE is the best, followed by RCD, OCBoost is the worst. The advantages of RDP are not obvious. The reason is the fact that the block-based ensemble classifier AWE is designed to cope mainly with gradual concept drifts.

**Figure 7:**
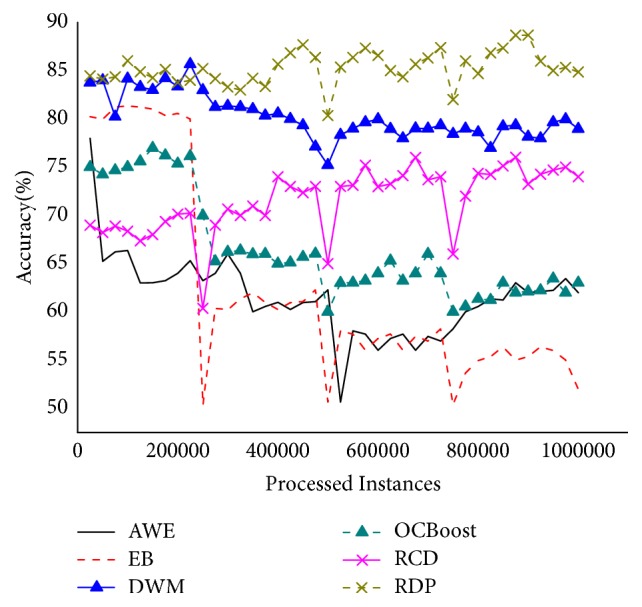
Accuracy on the Random Tree.

Figure 7 demonstrates the accuracy on the Random Tree, which is designed to evaluate the ability to handle sudden concept drifts. As can be seen, RDP is the best, followed by RCD, EB and AWE perform almost identically, with DWM being slightly less accurate, and OCBoost is the worst. Whenever a concept drift occurred, the accurate rates of all the algorithms will undergo instantaneous fluctuations except RDP, which maintains a high, stable accuracy and suffered the smallest accuracy drops. This might be attributed to the addition of drift detector which could capture concept drifts promptly and construct a new classifier to handle this type of drift.

**Figure 8:**
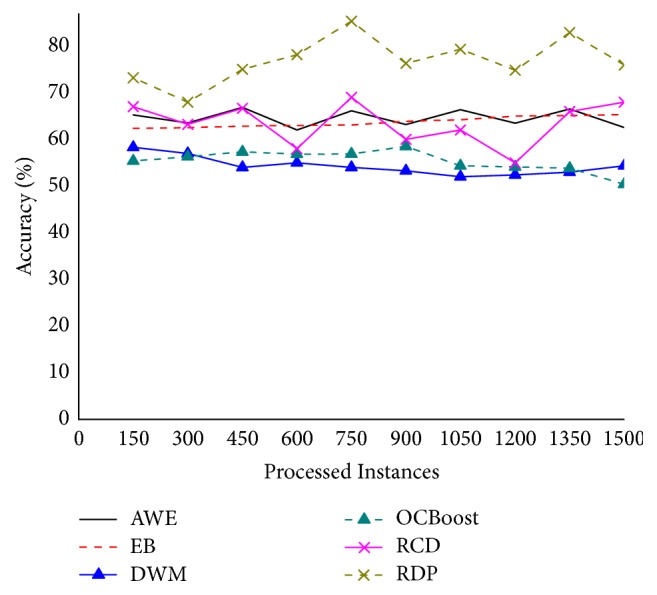
Accuracy on the Elist.

Figure 8 depicts the accuracy changes on the Elist. It can be observed that the accuracy curves of all algorithms are with varying degrees of volatility, which indicates that concept drift may exists in the dataset. Notice the sudden drops of all methods at drift time points (after 300, 600, 900, and 1,200 instances). However, RDP manages to recover faster in all cases exploiting and updating older models. RDP is the most accurate one, followed by the RCD. Unfortunately, DWM and OCBoost perform poorly on this dataset and the curves of them almost identically, while the accuracy curve of RDP is relatively stable, subjecting to data concept drift minimal impact on real data, which showed that the algorithm had better adaptability for this environment. This is partly because the management of the recurrent change detection mechanism is able to reuse historical concepts and achieve the better performance in different situations, particularly under concept drift environments.

**Figure 9:**
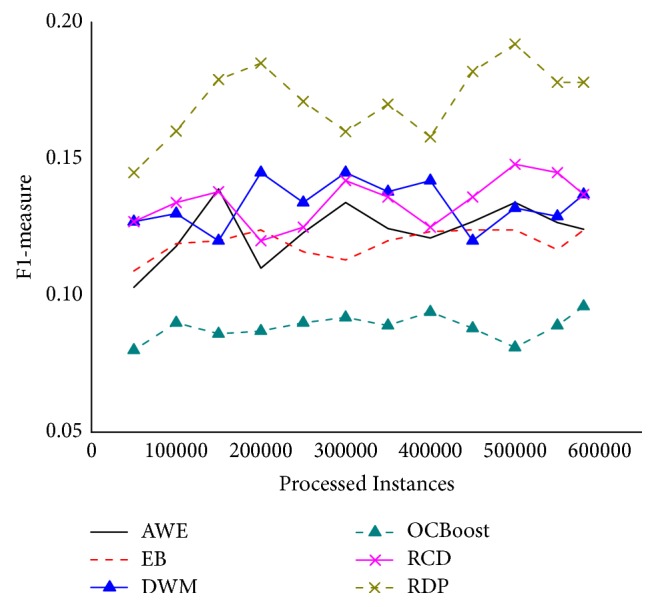
F_1_-measure on the Covertype.

Figure 9 shows the F_1_-measure changes on the Covertype. An interesting finding is that the curve of OCBoost suffered the most dramatic fluctuation. Apart from these, the F_1_-measure of RDP and RCD is relatively stable on this dataset. RDP gained the best performance on this dataset. It is also indicates that the algorithm has superior adaptability to the real stream environment.

**Figure 10:**
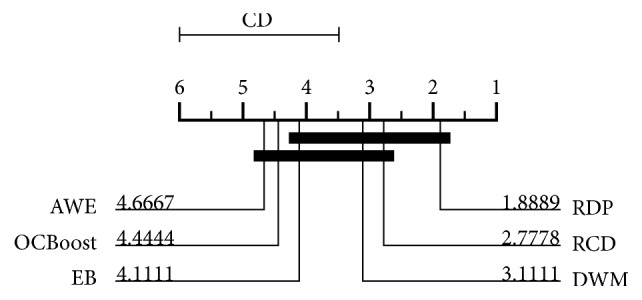
A critical different diagram for all classifiers against each other.

The average rank of classification accuracy of RDP on 9 synthetic and real-world data streams wins all other classifiers. To extend the analysis, nonparametric Friedman test was carried out for comparing multiple classifiers over multiple datasets [31]. The null hypothesis for the test is that there is no difference between the performances of all the tested algorithms. Moreover, in case of rejecting this null hypothesis, we employ the Nemenyi test [32] to verify whether the performance of our method is statistically different from the remaining algorithms. The critical difference diagram shown in Figure 10 tells that our method is significantly better than AWE and OCBoost.

To summarize, RDP is superior to the other three from the following aspects: (1) It is capable of constructing a satisfactory model for handling both sudden and gradual concept drifts and has been specifically built to deal with recurring concepts. (2) Compared to other ensembles, our method can achieve better performance and is robust against various noise levels and different types of drift.

## 5. Conclusion and Future Work

In this study, we focus on the investigation of Recurring Concept Drifts, a special subtype of concept drifts that has not yet drawn enough attention from the research community. A novel method intending to handle recurring concept based on the change detection method using classifier graph was proposed. The approach of detecting recurring drifts which allows reuse of previously learnt models enhanced the learning performance on most datasets. Extensive experiments on synthetic and real-world datasets have validated that the proposed approach not only outperforms the existing popular methods in the adaptation to Recurring Concept Drifts, but also adapts well even in different concept drifting scenarios.

In our ongoing and future research, we will explore an effective procedure which will eliminate redundant classifiers without decreasing the ability to deal with recurring concepts and work toward developing classifier graph based effective practical dynamical learning mechanism.

## Figures and Tables

**Algorithm 1:**
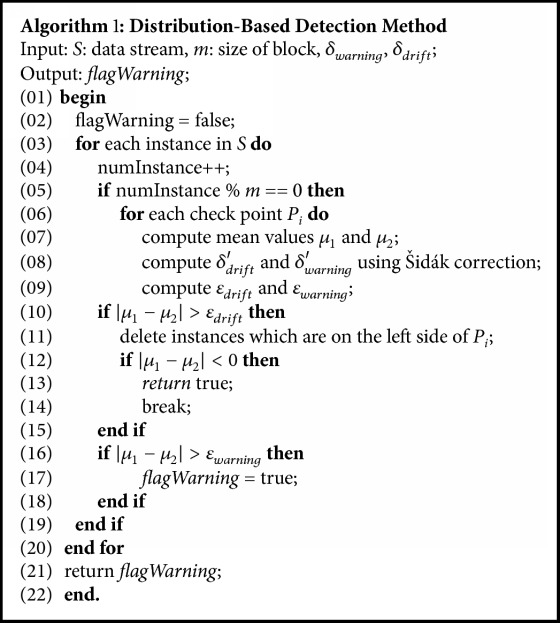
The pseudocode of DBDM.

**Algorithm 2:**
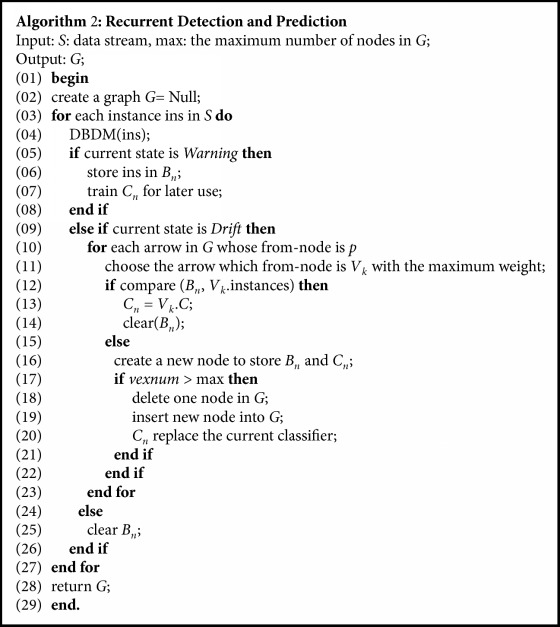
The pseudocode of RDP.

**Table 1:** Average false positive counts on stationary Bernoulli distribution.

	0.05	0.1	0.3	0.5
DDM	1.89	0.76	0.29	0.19
EDDM	35.56	36.3	14.42	9.38
ECDD	166.34	157.3	154.01	0.11
DBDM	3.39	0.95	0.05	0.03

**Table 2:** Average false positive counts on sudden concept drift.

mean value increment	0.04	0.09	0.29	0.49
DDM	11.93	10.06	8.34	8.7
EDDM	46.00	45.14	32.63	25.17
ECDD	173.81	169.55	168.07	91.43
DBDM	4.99	3.76	7.69	9.77

**Table 3:** Detection delays on an abrupt drift.

mean value increment	0.04	0.09	0.29	0.49
DDM	3823.96	1817.97	419.66	300.92
EDDM	1758.95	759.96	221.06	**148.59**
ECDD	**528.61**	497.99	522.97	426.8
DBDM	529.29	**235.64**	**200**	200

**Table 4:** Average false negative counts on a gradual drift.

Slope	0.0001	0.0002	0.0003	0.0004
DDM	0.75	0.58	0.51	0.32
EDDM	0.98	0.97	0.91	0.83
ECDD	0	0	0	0
DBDM	0	0	0	0

**Table 5:** Detection delays on a gradual drift.

Slope	0.0001	0.0002	0.0003	0.0004
DDM	-* *-	-* *-	-* *-	-* *-
EDDM	-* *-	-* *-	-* *-	-* *-
ECDD	523.55	508.5	515.58	509.25
DBDM	702	454	330	328

**Table 6:** Description of the nine datasets.

Dataset	Instances	Attributes	Drift type	Noise
HyperPlane	1M	10	gradual	5%
LED	1M	24	sudden	15%
Random Tree	1M	10	recurring	0%
SEA	1M	3	sudden recurring	10%
Elist	1, 500	913	recurring	-
Spam	9, 324	850	gradual	-
Usenet	5, 931	658	unknown	-
Covertype	581*K*	53	unknown	-
Gas Sensor	13, 910	128	unknown	-

**Table 7:** Characteristics of Elist.

	1-300	300-600	600-900	900-1200	1200-1500
Medicine	+	-* *-	+	-	+
Space	-* *-	+	-* *-	+	-* *-
Baseball	-* *-	+	-* *-	+	-* *-

**Table 8:** Comparison of classification accuracy (%).

	AWE	EB	DWM	OCBoost	RCD	RDP
HyperPlane	**86.98 (1)**	78.79 (4)	75.21 (5)	74.81 (6)	85.64 (2)	84.24 (3)
LED	59.94 (5)	53.48 (6)	**69.49 (1)**	62.65 (4)	67.65 (2)	66.89 (3)
Random Tree	65.17 (4)	66.53 (3)	61.25 (5)	53.67 (6)	67.53 (2)	**68.89 (1)**
SEA	72.01 (6)	77.60 (5)	**85.21 (1)**	83.89 (3)	81.45 (4)	84.65 (2)
Elist	54.08 (5)	65.36 (3)	55.07 (4)	51.45 (6)	67.89 (2)	**75.59 (1)**
Spam	67.27 (6)	70.13 (4)	**78.79 (1)**	72.79 (2)	69.19 (5)	71.36 (3)
Usenet	61.21 (6)	**79.58 (1)**	62.76 (5)	63.47 (4)	70.78 (3)	72.23 (2)
Covertype	73.24 (3)	66.86 (6)	70.63 (4)	69.63 (5)	78.53 (2)	**81.69 (1)**
Gas Sensor	56.45 (6)	57.07 (5)	64.30 (2)	59.45 (4)	62.36 (3)	**65.59 (1)**
Average Rank	4.67	4.11	3.11	4.44	2.78	**1.89**

**Table 9:** Comparison of time consumption (Cpu seconds).

	AWE	EB	DWM	OCBoost	RCD	RDP
HyperPlane	**13.47 (1)**	14.40 (2)	59.01 (5)	153.08 (6)	29.21 (3)	35.35 (4)
LED	20.98 (5)	31.36 (6)	**10.51 (1)**	12.43 (3)	19.98 (4)	11.24 (2)
Random Tree	37.42 (3)	38.18 (4)	49.53 (5)	78.13 (6)	31.42 (2)	**30.24 (1)**
SEA	36.41 (5)	44.56 (6)	20.01 (2)	**13.87 (1)**	26.41 (4)	24.05 (3)
Elist	17.36 (2)	33.45 (5)	50.21 (6)	22.23 (4)	20.11 (3)	**7.02 (1)**
Spam	81.43 (4)	63.01 (2)	85.23 (5)	86.36 (6)	**60.43 (1)**	80.25 (3)
Usenet	20.21 (2)	26.69 (3)	30.21 (5)	30.78 (6)	28.01 (4)	**16.69 (1)**
Covertype	26.41 (4)	13.43 (2)	36.06 (5)	41.56 (6)	**6.41 (1)**	14.67 (3)
Gas Sensor	86.57 (3)	90.12 (4)	97.79 (6)	96.41 (5)	80.12 (2)	**71.21 (1)**
Average Rank	3.22	3.56	4.44	4.78	2.67	**2.11**

**Table 10:** Comparison of memory consumption (MB).

	AWE	EB	DWM	OCBoost	RCD	RDP
HyperPlane	25.98 (5)	28.79 (6)	**10.21 (1)**	13.81 (2)	13.90 (3)	16.24 (4)
LED	29.90 (5)	39.90 (6)	20.34 (4)	**2.65 (1)**	13.78 (3)	6.89 (2)
Random Tree	45.17 (5)	78.53 (6)	18.28 (2)	34.07 (4)	23.07 (3)	**10.89 (1)**
SEA	412.01 (6)	343.30 (5)	**36.89 (1)**	68.65 (2)	73.12 (3)	78.21 (4)
Elist	80.35 (5)	89.45 (6)	**13.60 (1)**	34.82 (2)	44.48 (4)	42.58 (3)
Spam	25.98 (6)	24.79 (5)	20.01 (3)	22.36 (4)	**4.81 (1)**	16.24 (2)
Usenet	39.90 (6)	23.78 (5)	22.24 (4)	**2.65 (1)**	20.24 (3)	16.89 (2)
Covertype	45.17 (5)	78.53 (6)	**10.20 (1)**	23.07 (3)	10.89 (2)	26.25 (4)
Gas Sensor	412.01 (5)	543.30 (6)	410.21 (4)	376.89 (3)	326.07 (2)	**308.65 (1)**
Average Rank	5.33	5.67	**2.33**	2.44	2.67	2.56

**Table 11:** Comparison of F_1_-measure.

	AWE	EB	DWM	OCBoost	RCD	RDP
HyperPlane	**0.157 (1)**	0.097 (2)	0.068 (6)	0.079 (5)	0.086 (4)	0.094 (3)
LED	0.118 (6)	0.156 (5)	**0.280 (1)**	0.262 (3)	0.245 (4)	0.279 (2)
Random Tree	0.451 (3)	0.345 (4)	0.221 (5)	0.207 (6)	0.477 (2)	**0.489 (1)**
SEA	0.089 (6)	0.127 (5)	0.141 (4)	0.189 (3)	**0.247 (1)**	0.235 (2)
Elist	0.098 (3)	0.079 (4)	0.069 (5)	0.058 (6)	0.156 (2)	**0.224 (1)**
Spam	0.027 (6)	**0.313 (1)**	0.169 (4)	0.079 (5)	0.248 (2)	0.216 (3)
Usenet	0.039 (5)	0.033 (6)	**0.078 (1)**	0.047 (3)	0.057 (2)	0.043 (4)
Covertype	0.120 (5)	0.127 (4)	0.136 (3)	0.073 (6)	0.147 (2)	**0.169 (1)**
Gas Sensor	0.046 (4)	0.037 (6)	0.055 (3)	0.045 (5)	0.047 (2)	**0.059 (1)**
Average Rank	4.33	4.11	3.56	4.67	2.33	**2.00**
